# Flat and roll-type translucent anodic porous alumina molds anodized in oxalic acid for UV nanoimprint lithography

**DOI:** 10.1039/d3ra06240f

**Published:** 2023-11-13

**Authors:** Takashi Yanagishita, Naoko Kumagai, Hideki Masuda

**Affiliations:** a Department of Applied Chemistry, Tokyo Metropolitan University 1-1 Minamiosawa, Hachioji Tokyo 192-0397 Japan yanagish@tmu.ac.jp

## Abstract

There is much interest in UV nanoimprinting as a fabrication method for various functional devices because of its suitability for efficient fine patterning. To form patterns on opaque substrates by UV nanoimprinting, it is essential to use molds through which UV light can pass. In this study, translucent anodic porous alumina (APA) molds for UV nanoimprinting were fabricated by the anodization of an Al substrate. To fabricate a translucent APA mold, an ordered APA film used as a mold for UV nanoimprinting was formed on the surface side of the Al substrate, and then anodization was continued from the back surface of the Al substrate to increase its transparency in the UV spectral range. A gradient change of Al thickness is necessary for the production of a large-area translucent mold, since it lowers the thickness of opaque defects remaining in the mold. The resulting translucent mold was effective for UV nanoimprinting to prepare ordered polymer nanopillar arrays on the surfaces of opaque substrates because the transmittance of the resulting translucent APA mold was 40% at a wavelength of 365 nm, which was confirmed to be sufficiently translucent to polymerize the photocurable monomer used in this study. In addition, it was possible to fabricate roll-type translucent APA molds by using Al pipes as a starting material. A seamless ordered nanopillar array can be effectively formed on a substrate by continuous UV nanoimprinting using the resulting roll-type translucent APA molds. Ordered nanopillar arrays formed on opaque substrates by UV nanoimprinting using translucent APA molds have various potential applications, such as those for forming antireflective and water-repellent surfaces.

## Abstract 2

Transparent anodic porous alumina mold for photo-nanoimprinting.

## Graphical abstract

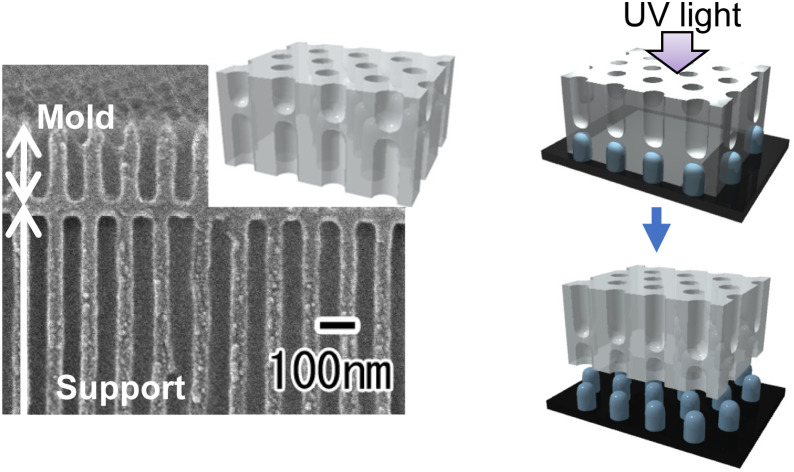

## Introduction

Ultraviolet (UV) nanoimprinting is a promising method for fabricating various functional devices, such as optical devices, biodevices, and sensors, because ordered patterns can be efficiently formed on a substrate surface by this method using a mold and a photocurable monomer.^1–12^ In this process, UV light is irradiated on the photocurable monomer between the mold and the substrate for curing, and then the mold is peeled off from the resulting cured polymer to form an ordered pattern of the polymer on the substrate. The ordered patterns obtained by UV nanoimprinting are inverted structures of the surface structure of the nanoimprinting mold. Therefore, to fabricate ordered patterns suitable for various applications by UV nanoimprinting, it is important to develop a mold preparation process that allows precise pattern control and easy scale-up. The molds used for UV nanoimprinting have usually been prepared by a combination process of electron beam lithography and dry etching.^13–15^ This method enables the formation of various ordered patterns on a mold surface; however, it also has the disadvantages of requiring expensive equipment and the difficulty of preparing large-area ordered patterns, which limit the range of applications of UV nanoimprinting. The fabrication of nanoimprinting molds using a self-assembling method that enables the fabrication of large-area, ordered patterns with inexpensive equipment has also been investigated.^16–18^ For example, the fine-particle lithography technique, which uses a self-organized regular array of particles as a mask to etch substrates, enables the formation of large-area, regular patterns.^19^ However, the polymer or silica particles used to form the ordered particle arrays are easily etched during the dry etching of a substrate, making it difficult to form high-aspect-ratio structures on mold surfaces through particle lithography.

We have been investigating UV nanoimprinting using anodic porous alumina (APA), which is a self-organized nanohole array material prepared by anodizing Al in an electrolyte, as a mold.^20–27^ APA is a promising material as a nanoimprinting mold for forming ordered nanopillar arrays with high aspect ratios because it is possible to obtain ordered nanohole array structures with high aspect ratios by anodizing Al under appropriate anodization conditions.^28^ In our previous papers, we have also reported that the resulting nanopillar arrays are useful for fabricating various functional devices such as photonic crystals, antireflection structures, and superhydrophobic surfaces.^29–31^ The problem with the APA molds for UV nanoimprinting is their limited use for translucent substrates because they are opaque; thus, UV irradiation from the mold side is not possible. If the translucentization of APA molds can be achieved, UV irradiation from the mold side becomes possible and APA molds can be applicable to opaque substrates, thereby contributing to the expansion of the range of applications of nanoimprinting using APA molds. Several methods for producing non-opaque APA films have been reported thus far.^32–37^ However, the resulting APA films have not been used as molds for UV nanoimprinting. Because the height of the polymer pillar array obtained by UV nanoimprinting corresponds to the pore depth of the APA mold, it is necessary to control the pore depth of the APA mold to control the height of the resulting polymer pillars. However, in the case of previously reported methods, when an APA film is thinned to form shallow pore molds, the film does not have a sufficiently high mechanical strength for use as a mold for nanoimprinting. Although a method of fabricating thin APA masks on a substrate has been reported, it is difficult to fabricate large-area APA masks, and the adhesion strength between the APA mask and the substrate is low, making it difficult to use them as molds for nanoimprinting.^38^ Translucent APA with shallow pores can also be fabricated by anodizing the Al film deposited on a transparent conducting glass substrate, such as indium thin oxide (ITO) glass or fluorine-doped thin oxide (FTO) glass.^39,40^ However, the low adhesion strength between APA and the substrate makes it difficult to use translucent APA formed on the substrate as a mold for nanoimprinting. Therefore, it has been difficult to fabricate translucent APA that can be used as a mold for nanoimprinting using previously reported APA fabrication methods.

In this report, we describe the fabrication of translucent APA molds. Here, the translucentization of APA molds was achieved by anodizing the entire residual Al in the substrate of the molds, enabling UV transmission through the anodized back surface. This is the first report on the fabrication of translucent APA molds for UV nanoimprinting by the anodization of all Al substrates. In this study, the fabrication of not only flat translucent APA molds but also roll-type translucent molds was investigated. The translucent APA molds obtained in this study are useful as UV nanoimprinting molds for the efficient fabrication of ordered patterns on opaque substrates.

## Experimental section

**Fig. 1:**
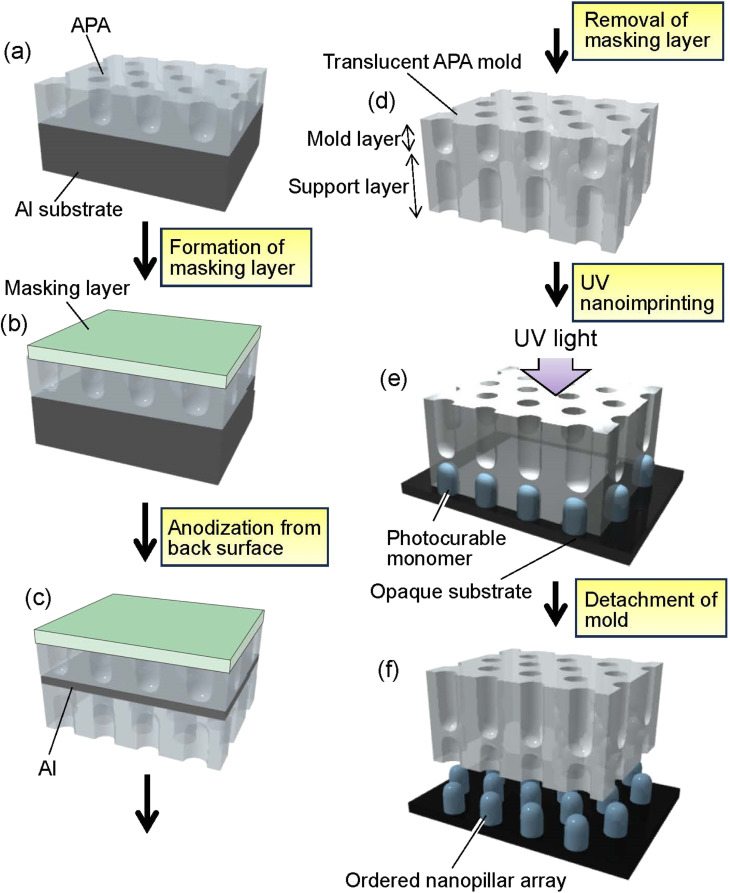
Schematic of preparation process for translucent APA mold and UV nanoimprinting using the resulting translucent mold; (a) formation of mold layer by anodization, (b) formation of masking layer on the surface of mold layer, (c) anodization of the sample to prepare a support layer, (d) removal of masking layer to obtain translucent APA mold, (e) UV nanoimprinting, and (f) detachment of the APA mold to form ordered polymer nanopillar array.

Fig. 1 shows a schematic of the preparation process for translucent APA molds. In this study, experiments were conducted using two types of Al sheet (99.99% purity). One is an Al sheet with a size of 5 × 2 cm^2^ and a thickness of 400 μm. The other is an Al sheet with a size of 5 × 2 cm^2^, whose thickness increased in a gradient from 100 to 300 μm. The thickness of the Al sheet was processed to vary linearly and continuously. Al sheets were electropolished in a mixture of 20 vol% perchloric acid and 80 vol% ethanol at 0 °C under a constant current of 0.2 A cm^−2^ for 4 min. For the preparation of self-organized APA with an ordered hole arrangement, a two-step anodization process reported previously was employed.^41^ The electropolished Al sheets were anodized at 40 V in 0.3 M oxalic acid at 17 °C for 17 h using the wide-range DC power supply (PSW-720H800Y1H, Texio Technology Co., Japan). In this study, an Al sheet was also used as the counter electrode, and a cylindrically rounded Al sheet was placed around the inner wall of a 500 ml glass beaker used for the anodization. The distance between the sample and the counter electrode was adjusted to 2 cm. The anodized oxide layer was completely dissolved by immersing an Al sheet in a mixture of 6 wt% phosphoric acid and 1.8 wt% chromic acid at 70 °C for 3 h to obtain an Al sheet with an ordered dimple array on its surface corresponding to the pore arrangement on the back surface of the dissolved APA. The resulting Al sheet was anodized at 40 V in 0.3 M oxalic acid at 17 °C for 90 s to form a thin APA that acts as a mold for UV nanoimprinting (Fig. 1(a)). After the formation of the mold layer, the pore size of the obtained APA was increased by etching with 5 wt% phosphoric acid at 30 °C for 20 min to adjust the diameter of the polymer pillar arrays obtained by nanoimprinting. To fabricate a translucent APA mold, a rubber-based masking agent (Sunecon Mask Ace S, Taiyo Chemical & Engineering Co., Ltd., Japan) was applied to one side of a sample to form a masking layer (Fig. 1(b)), and the sample was reanodized in 0.5 M oxalic acid at 5 °C at a constant voltage of 40 V for about 6 days until the sample became translucent (Fig. 1(c)). The anodization to make the samples translucent was performed at a low dissolution rate of the formed APA and a high film growth rate. In this study, the anodization of the residual Al was performed under conditions similar to those of mild anodization, rather than hard anodization, in which a high current density is applied. The growth rate of the support layer was not investigated in detail in this study because we found from our preliminary studies that the growth rate of APA is not constant for long periods of time during anodization, but the growth rate of APA generally increases with the electrolyte concentration. In addition, decreasing the electrolyte temperature suppresses the dissolution of the APA, thus preventing the increase in pore size even after a long anodization period. From results of preliminary studies, anodization conditions with a high film growth rate and a low pore diameter increase rate were adopted in this study for preparing the support layer. After the anodization, the masking layer was dissolved with toluene to obtain a translucent APA mold because the diluent for the masking agent used in this study was toluene (Fig. 1(d)).

The surfaces of APA molds were modified with fluoroalkylsilane (Optool DSX, Daikin Industries, Ltd., Japan) to form a release layer prior to UV nanoimprinting. In the nanoimprinting process, a release layer is formed on the mold surface to facilitate the detachment of the mold from the polymer fine patterns. Generally, silicone or fluorinated surface modifiers are used to form the mold release layer. In this study, fluoroalkylsilane was used as the release layer on the APA mold surface. A commercially available photocurable monomer (PAK-02, Toyo Gosei Co., Ltd., Japan) was used to form ordered polymer nanopillar arrays by UV nanoimprinting (Fig. 1(e)). After dropping the photocurable monomer onto a Si wafer, a translucent APA mold was placed onto the substrate and irradiated with UV light using UV-light-emitting diodes (UV-LEDs) with a center wavelength of 365 nm through the alumina mold for 2 min in N_2_ atmosphere to solidify the monomer. The translucent APA mold was peeled off from the solidified polymer layer to obtain ordered polymer nanopillar arrays on the Si substrate (Fig. 1(f)). The obtained samples were observed by field emission scanning electron microscopy (FE-SEM; JSM-6700F, JEOL). The vacuum in the sample chamber of SEM was adjusted to less than 5.0 × 10^4^ Pa, and SEM observations were performed at an accelerating voltage of 5 kV. All SEM images of the APA and the polymer nanopillar arrays in this report were observed under the same conditions. The transparency of the obtained molds was evaluated using a spectrophotometer (Solidspec-3700, Shimadzu). Transmission spectra of the samples were measured in the range of 350–800 nm at a scan rate of 5 nm s^−1^.

## Results and discussion

**Fig. 2:**
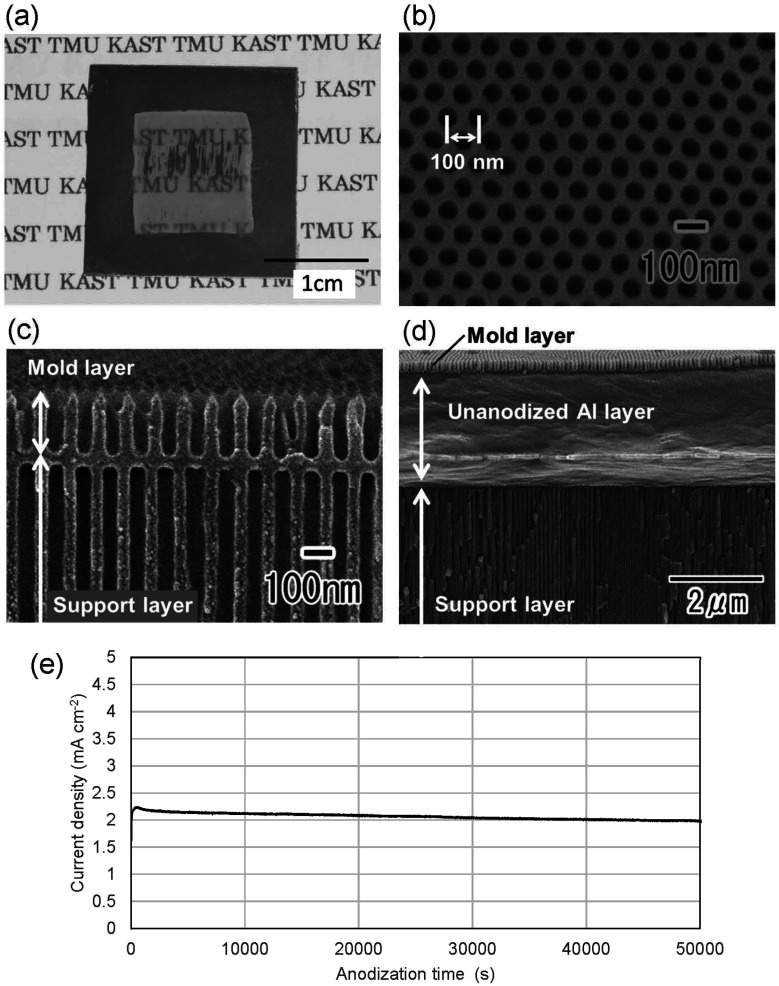
(a) Photograph of translucent APA mold. (b) Surface and (c) cross-sectional SEM images of the translucent APA mold. (d) Cross-sectional SEM image of the opaque portion of the APA mold. (e) Time–current density curve of the sample during the anodization for fabricating the support layer.

Fig. 2(a) shows a photograph of a translucent APA mold prepared by anodizing Al. In this sample, after forming an ordered APA that serves as a mold, the entire surface and the peripheral areas of the back surface were masked before anodization, resulting in an APA mold with only the central area being translucent. From the photograph taken after anodization shown in Fig. 2(a), it can be observed that most of the unmasked areas have become translucent. The partially opaque parts are areas where unoxidized Al remained during the long anodization from the back surface of the sample. In this process, once all the surrounding Al is anodized, the remaining Al is not anodized any further and remains as opaque defects because electricity cannot be applied to the remaining Al. That is, if the remaining Al cannot be energized, no further anodization will proceed, and Al will remain between the mold and the support layer. Residual fraction of Al substrate with a thickness of 100 nm or more is almost impervious to UV light and cannot be used as translucence APA molds if more than 100 nm of residual Al remains between the mold and the support layer.^42^Fig. 2(b) and (c) show the surface and cross-sectional SEM images of the resulting translucent APA mold. As shown in Fig. 2(b), uniformly sized pores were observed to be orderly arranged over the entire surface of the sample. The interpore distance and pore diameter of the obtained APA were 100 and 60 nm, respectively. From the cross-sectional SEM image shown in Fig. 2(c), an APA film that functions as a support layer is formed beneath an ordered APA film with a pore depth of 220 nm that functions as a mold. Although the pores in the mold and the support layer are observed almost on the same line, this is coincidental. It was also confirmed that no unoxidized Al remained between the two APA layers in the completely translucent areas. In addition, it was confirmed that if an unoxidized Al layer remains between the porous alumina layers, as shown in Fig. 2(d), the areas with the Al layers remain as opaque defects. It was also confirmed that the mask was completely dissolved and removed from the APA surface by toluene. Fig. 2(e) shows the time–current density curves of the anodization for fabricating the support layer, which confirmed that the current density was low, and the anodization conditions were mild. At the air–electrolyte interface, the electrolyte spread wetting to the upper side of the Al sheet, so that even after 6 days of anodization, a slight anodization reaction continued at the top of the sample. Therefore, whether or not the sample became translucent was determined visually, not by current density.

**Fig. 3:**
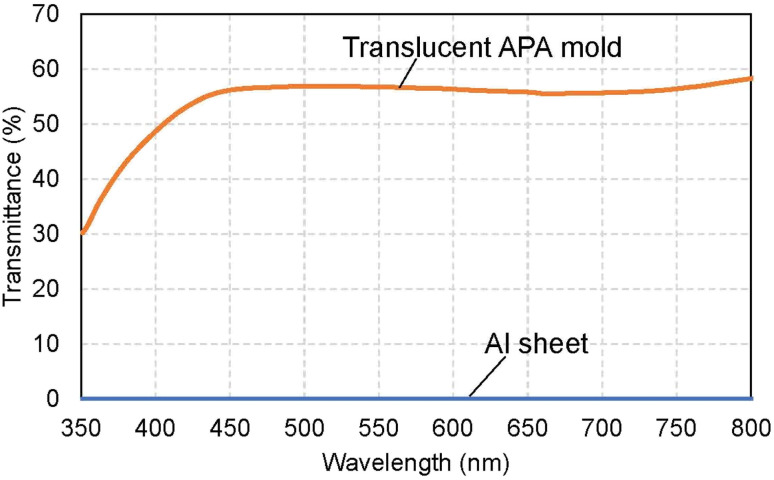
Transmittance spectra of the translucent APA mold and Al sheet with a thickness of *ca.* 300 μm.

Fig. 3 shows the transmittance spectrum of the translucent APA mold obtained by this process. The thickness of the resulting APA mold used for transmittance measurement was 300 μm. For comparison, the transmittance spectrum of an Al sheet with a thickness of *ca.* 300 μm is also shown in Fig. 3. It is observed in Fig. 3 that the resulting APA mold exhibited a transmittance of about 55% over the entire visible light wavelength range. The APA films prepared using an oxalic acid electrolyte appear yellowish owing to the oxalic acid anions incorporated into the films, and the decrease in transmittance at wavelengths shorter than 450 nm may be due to the oxalic acid anions incorporated into the films.^43,44^ For the nanoimprinting, a UV-LED with a center wavelength of 365 nm was used to cure the photocurable monomer. The transmittance of the resulting translucent APA mold was 40% at 365 nm, which was confirmed to be sufficiently translucent to polymerize the photocurable monomer used in this study. In this process, the anodization conditions for residual Al for the formation of the support layer need not match those for the mold layer, and the translucency of the sample can also be achieved by anodization with sulfuric or phosphoric acid. The main objective of this study was not to maximally increase the transparency of the mold but to make the mold sufficiently translucent to allow UV light to penetrate so that the photocurable monomer can be cured. Therefore, in this study, to simplify the experimental process, the mold and support layers were formed by anodizing Al with the same type of electrolyte at the same voltage.

**Fig. 4:**
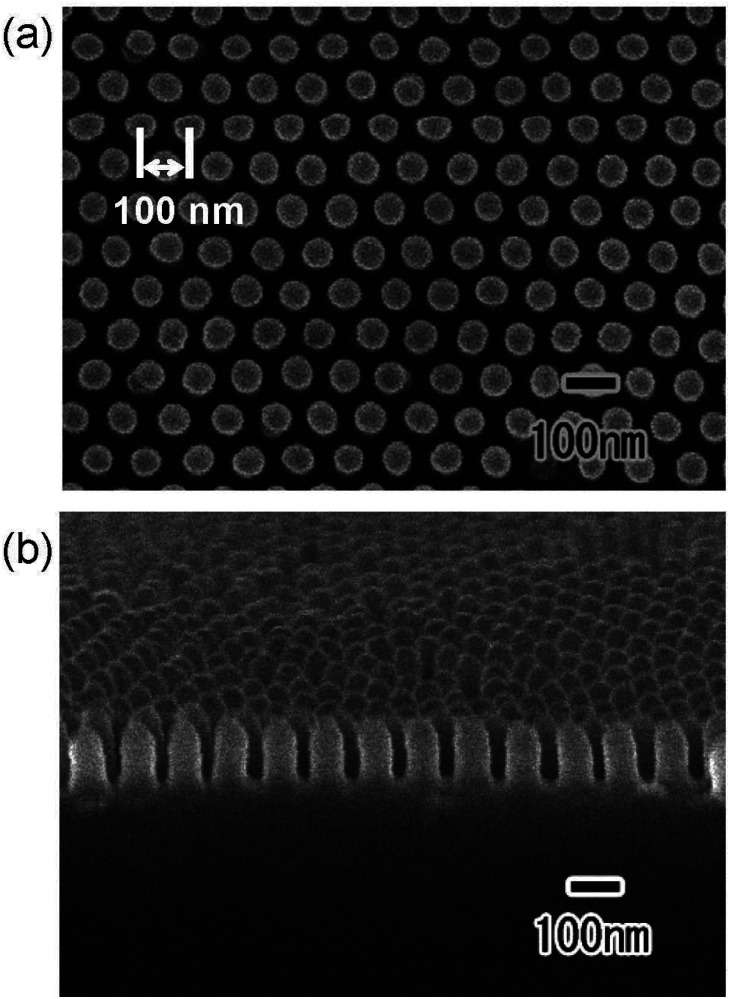
(a) Surface and (b) cross-sectional SEM images of polymer nanopillar array formed on the surface of Si substrate by UV nanoimprinting using translucent APA mold.

Fig. 4 shows the (a) surface and (b) cross-sectional SEM images of a polymer nanopillar array fabricated on a Si substrate by UV nanoimprinting using the translucent APA mold. UV light was irradiated for UV nanoimprinting using the translucent APA mold. An ordered structure of uniformly sized nanopillars, corresponding to the surface structure of the translucent APA mold used for UV nanoimprinting, was obtained. The period of the obtained nanopillar array was 100 nm, which is the same as the interpore distance of the translucent APA mold used for UV nanoimprinting. The average diameter of the obtained polymer nanopillars was also 60 nm. This result shows that the polymerization and solidification of the photocurable monomer can be achieved by UV light irradiation through the translucent APA mold fabricated by this process and that the ordered fine patterns corresponding to the surface structure of the mold can be formed on opaque substrate surfaces. In the areas where several millimeter squares or more of unanodized Al remained, the photocurable monomer was not cured because UV light did not penetrate through these areas, and no nanopillar arrays were formed. However, in areas where translucency increased, defect-free regular pillar arrays were formed over a large area. This may be due to the following UV light diffused from the translucent areas to the areas where a small amount of unoxidized Al remained, thereby curing the photocurable monomer, or the polymerization reaction progressed in a chain reaction to the unirradiated areas. The aspect ratio of the resulting polymer nanofibers depends on the pore size and depth of the anodic porous alumina mold used for nanoimprinting. In our previous study, polymer nanofibers with an aspect ratio of 120 have been formed by nanoimprinting using an anodic porous alumina mold with a high aspect ratio.^24^

**Fig. 5:**
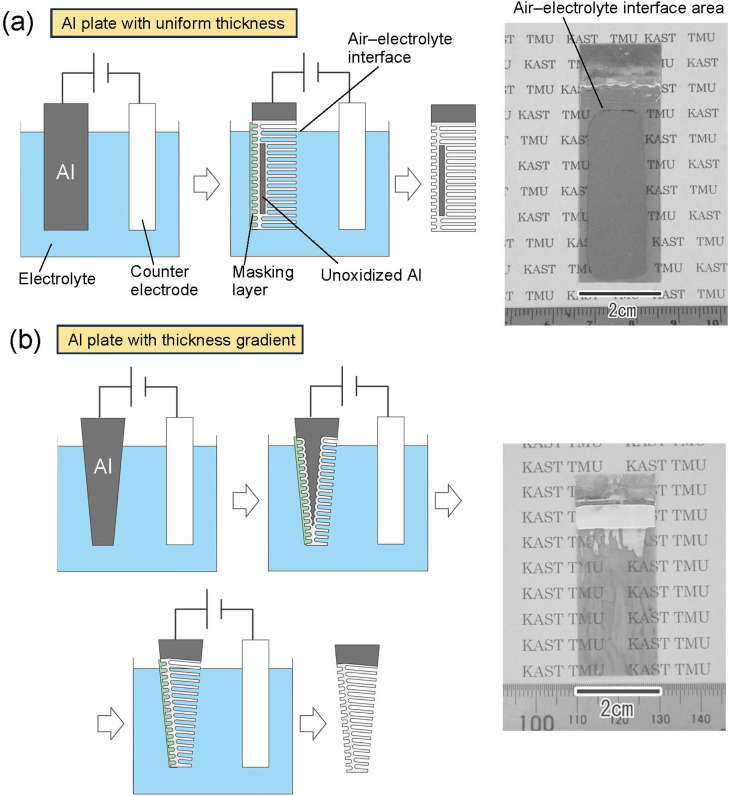
Schematic of the preparation process and photograph of samples obtained by anodization of (a) Al plate with uniform thickness and (b) Al plate with thickness gradient.

Fig. 5 shows the results of an attempt to fabricate a large translucent APA mold. Fig. 5(a) shows the results of anodization using an Al sheet with a uniform thickness of 400 μm as the starting material. The area of Al immersed in the electrolyte was adjusted so that the area of Al to be anodized was 5 × 2 cm^2^. After anodization, only the edges of the sample and the area corresponding to the air–electrolyte interface during anodization became translucent, but most areas of the sample remained opaque owing to the remaining unoxidized Al substrate. Since electrochemical reactions are usually more active at the electrode edges, the transparency of the sample is considered to have increased from the edges of the sample. The air–electrolyte interface of the Al substrate is not immersed completely in the electrolytic solution during anodization, so the reaction heat cannot be efficiently removed, and the oxide film growth rate is higher in the interface than in other areas. Therefore, the air–electrolyte interface of the sample become translucent ahead of the other areas of the sample. After the entire Al at the air–electrolyte interface was oxidized and became translucent, the Al remaining below the air–electrolyte interface could not continue to be energized; thus, most areas of the sample remained opaque. Therefore, it is considered that the increased translucency at the top and bottom of the sample is due to different factors, resulting in a different growth rate and a different area of the sample where translucency occurs. Insulating the edges of the Al sheet and the air–electrolyte interface with polymer non-conductive paste would prevent current from concentrating in those areas of the sample during anodization. As a result, it is possible to make the center of the sample translucent, as shown in Fig. 2(a). However, when the thickness of the Al sheet is uniform, partially unoxidized defects remain. Fig. 5(b) shows the results of fabricating a translucent APA mold using an Al plate with a thickness gradient as the starting material. In this experiment, an Al plate with a size of 5 × 2 cm^2^, whose thickness increased in a gradient from 100 to 300 μm, was fabricated by machining and used for anodization. The Al plate was anodized by immersing it in an electrolytic solution with the thinner side at the bottom and the thicker side at the top. From the photograph of the sample shown in Fig. 5(b), transparency was observed to have increased in most areas of the sample. This result shows that for an Al substrate with an anodization area of 4 × 2 cm^2^, a thickness gradient from 100 to 260 μm makes a large area transparent, with transparency increasing across the increasing thickness gradient. In this experiment, Al sheets with a thickness gradient were prepared to all anodized Al substrates. Using an Al plate with a gradient thickness as a starting material, we can complete the anodization of Al from the thin area, making it possible to achieve the translucency of the Al sheet over a large area. Although the relationship between APA film thickness and transmittance was not investigated in this study, the results of UV nanoimprinting using the APA shown in Fig. 5(b) as a mold confirmed that nanopillar arrays can be formed on the entire surface of the mold, regardless of the thickness gradient.

**Fig. 6:**
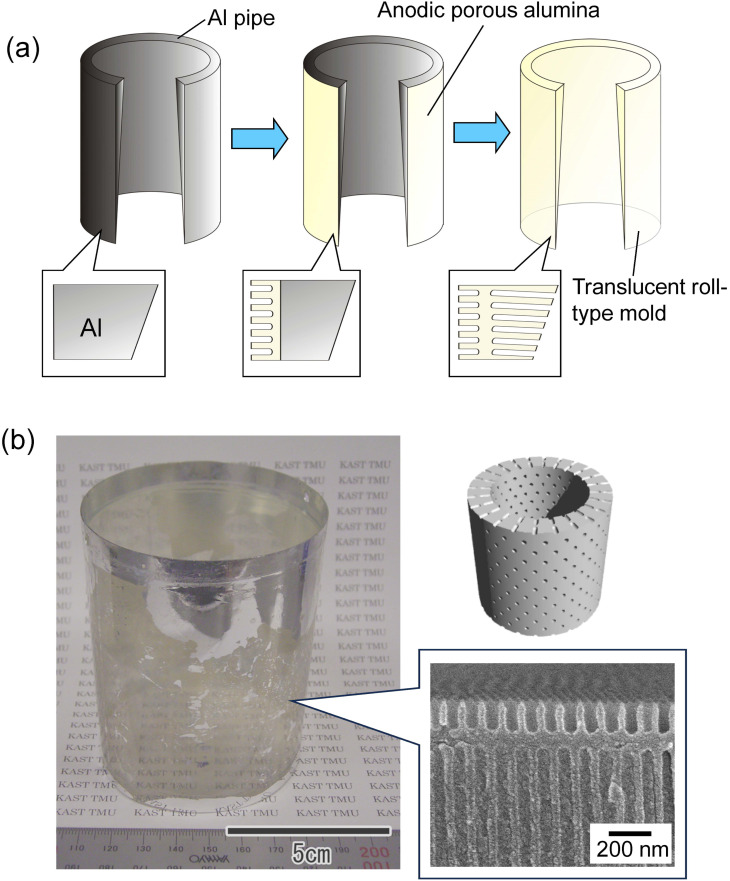
(a) Schematic of preparation of roll-type translucent APA mold. (b) Photograph and cross-sectional SEM image of the resulting roll-type translucent APA mold.

Fig. 6(a) shows a schematic of the preparation of a translucent roll-type mold using an Al pipe with a thickness gradient. The Al pipe with a diameter of 7 cm, a height of 9 cm, and a thickness gradient from 100 to 300 μm was used in this experiment. As with the flat Al plate, a thin porous alumina layer that functions as a nanoimprinting mold layer was formed on the periphery of the Al pipe. A masking agent was applied to the periphery of the pipe, and anodization was continued in 0.5 M oxalic acid at 5 °C at a constant voltage of 40 V until the pipe became translucent, as shown in Fig. 6(a). Fig. 6(b) shows the photograph and cross-sectional SEM image of the resulting translucent roll-type APA mold after the removal of the masking layer. Although there are some opaque areas due to Al remaining in some portions, transparency is observed to be high in most areas of the roll-type mold.

**Fig. 7:**
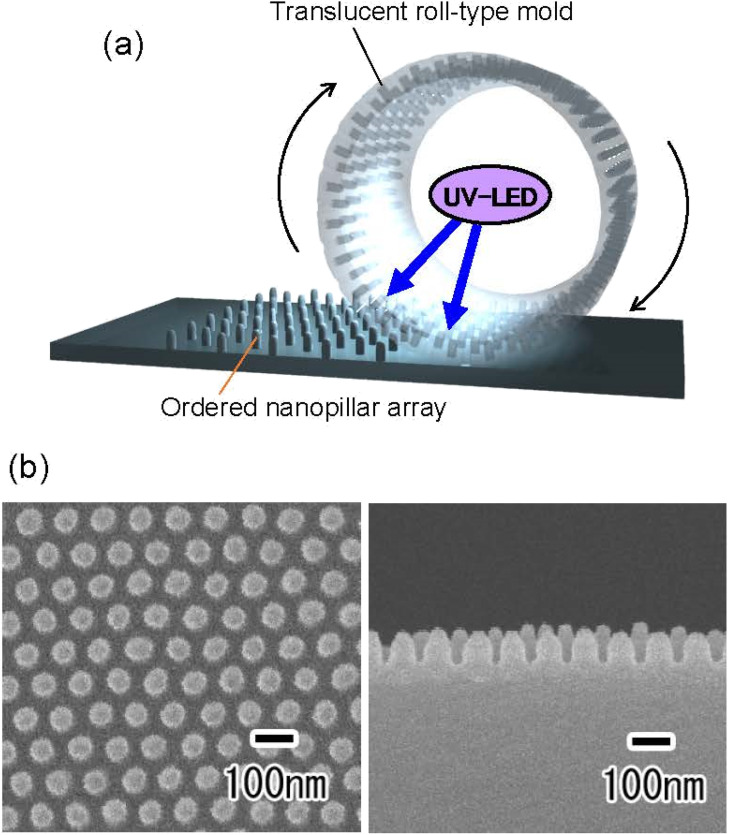
(a) Schematic of continuous UV nanoimprinting using roll-type translucent APA mold. (b) Surface and cross-sectional SEM images of polymer nanopillar array obtained by continuous UV nanoimprinting using roll-type translucent APA mold.

Fig. 7 shows the results of continuous UV nanoimprinting using the resulting translucent roll-type mold. To perform continuous UV nanoimprinting, a glass pipe was attached to the inside of the roll-type APA mold as a support. The roll-type mold support used in this experiment was a 1 mm-thick synthetic quartz glass pipe, which has been confirmed to transmit more than 90% of light at a wavelength of 365 nm. A polyethylene terephthalate sheet with a thickness of 0.5 mm was used as the substrate, and a UV-LED was placed inside the roll-type mold to solidify the photocurable monomer during UV nanoimprinting, as shown in Fig. 7(a). Fig. 7(b) shows the SEM images of the surface and cross section of the polymer nanopillar array obtained by continuous UV nanoimprinting while rotating the roll-type mold at a speed of 50 mm min^−1^. The orderly arranged array of uniformly sized nanopillars at intervals of 100 nm was obtained. Although further optimization of preparation conditions, such as Al pipe thickness control and anodization conditions, is necessary to fabricate translucent roll-type molds without opaque defects, this process has been shown to be applicable to forming translucent roll-type molds for continuous UV nanoimprinting. The Al pipe used in this experiment was machined from a block of 99.99% pure Al. Because high-purity Al is a very soft metal, it is difficult to machine, making it difficult to fabricate Al pipes with a thickness of less than 1 mm or with a gradient in thickness. As in the case of flat plates, we believe that translucency can be achieved over a large area if Al pipes with precisely controlled thickness are used as the starting material. The resulting roll-type mold has seamlessly formed ordered APA on its surface, making it possible to form a seamless ordered nanopillar array on an opaque substrate surface by continuous UV nanoimprinting. The obtained nanopillar arrays are expected to be applied to antireflective coatings and water-repellent surfaces.

## Conclusions

Translucent APA molds for UV nanoimprinting were fabricated by the anodization of Al substrates. In the present process, an ordered APA thin film, which serves as a mold for UV nanoimprinting, is first formed, followed by a masking layer on the surface of the mold layer; and finally, residual Al is anodized from the back surface of the sample. In the area where the masking layer is formed, neither the growth of the oxide film nor the increase in pore size by the dissolution of the film in an electrolyte progresses. Thus, transparency can be achieved while maintaining the structure of the porous alumina layer that functions as a nanoimprinting mold. In this study, we found that using an Al plate with a thickness gradient as a starting Al substrate contributes to the formation of large-area translucent APA molds, because transparency increases from the thin areas of the sample. Using an Al pipe as the starting material, we were able to fabricate a roll-type translucent APA mold. Seamless nanopillar arrays with an ordered arrangement can be efficiently fabricated on opaque substrate surfaces by continuous UV nanoimprinting using the roll-type translucent mold. The ordered nanopillar arrays fabricated by this process are expected to have various applications, such as those for forming antireflective and water-repellent surfaces.

## Author contributions

Takashi Yanagishita: conceptualization, methodology, writing of original draft. Naoko Kumagai: investigation. Hideki Masuda: supervision, manuscript-review and editing.

## Conflicts of interest

There are no conflicts to declare.

## Supplementary Material
